# Supplementary material for: Urinary Excretion of Iohexol as a Permeability Marker in a Mouse Model of Intestinal Inflammation: Time Course, Performance and Welfare Considerations
Source: Animals (Basel). 2021 Jan 4;11(1):79. doi: 10.3390/ani11010079 (PMC7824797; doi:10.3390/ani11010079)
Supplement: Supplementary file 1 [file animals-11-00079-s001.pdf]

animals

MDPI

Supplementary Materials: Urinary Excretion of Iohexol as a Permeability Marker in a Mouse Model of Intestinal Inflammation: Time Course, Performance and Welfare Considerations

Victoria Ortín-Piqueras [1,2,*,†], Tobias L Freitag [3,†], Leif C Andersson [4], Sanna H Lehtonen [4,5], Seppo K Meri [3], Thomas Spillmann [1] and Rafael Frias [2]

1 Department of Equine and Small Animal Medicine, Faculty of Veterinary Medicine, University of Helsinki, FIN-00014 Helsinki, Finland; thomas.spillmann@helsinki.fi
2 Comparative Medicine, Karolinska Institute, SE-171 77 Stockholm, Sweden; rafael.frias@ki.se
3 Translational Immunology Research Program, University of Helsinki, FIN-00014 Helsinki, Finland; tobias.freitag@helsinki.fi (T.L.F.); seppo.meri@helsinki.fi (S.K.M.)
4 Department of Pathology, University of Helsinki, FIN-00014 Helsinki, Finland; leif.andersson@helsinki.fi (L.C.A.); sanna.h.lehtonen@helsinki.fi (S.H.L.)
5 Research Programme for Clinical and Molecular Medicine, University of Helsinki, FIN-00014 Helsinki, Finland
* Correspondence: victoria.ortinpiqueras@helsinki.fi
† These authors share first authorship.

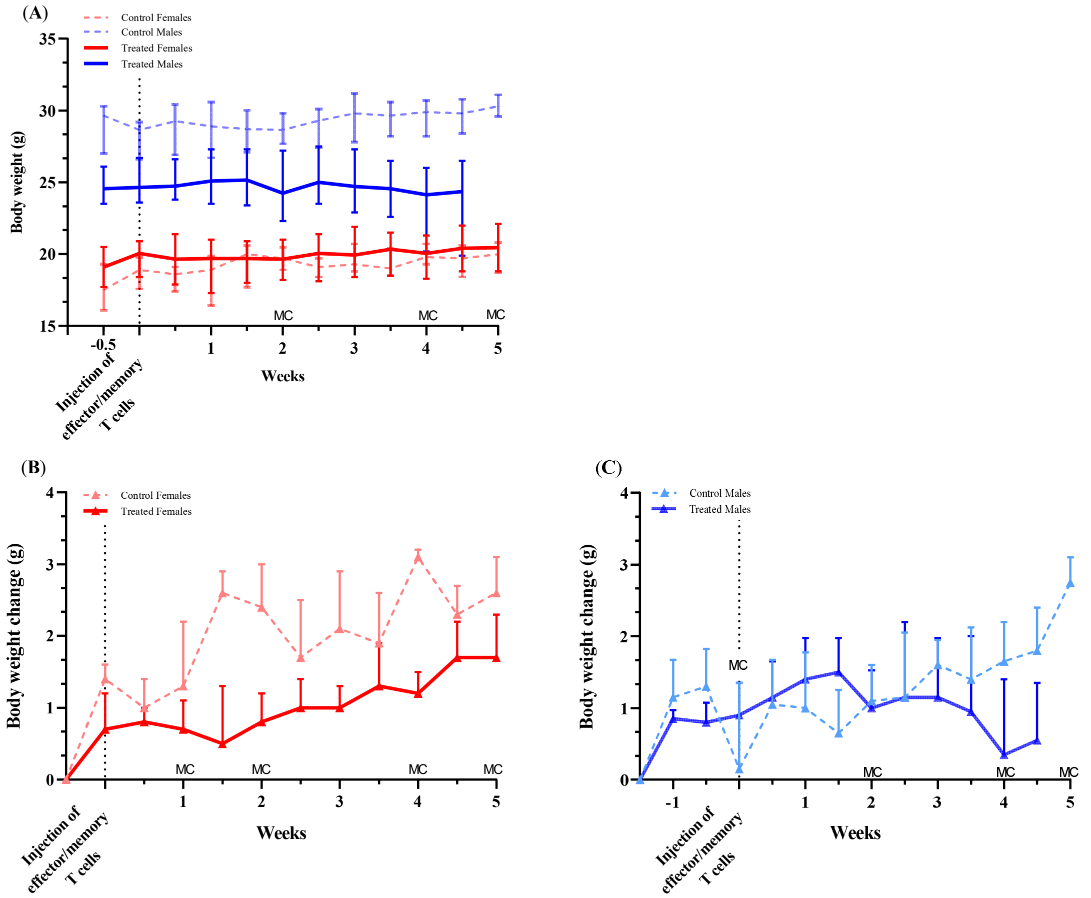

Figure S1. Effects of T cell injection and metabolic cage housing on body weight in mice. (**A**) Median ± IQR body weight (g) in control and treated animals over the length of the study. Effector/memory T cells were

injected in day zero. In W2, W4, and W5 intestinal permeability (IP) tests were performed in treated mice. There is not weight data for treated male mice on W5. Week 0 and W1 represent the performance of the IP testing in control male and female mice, respectively. Median ± IQR body weight changes (g) in control and treated (**B**) female or (**C**) male mice. *p < 0.05; **p < 0.001, ***p < 0.0001

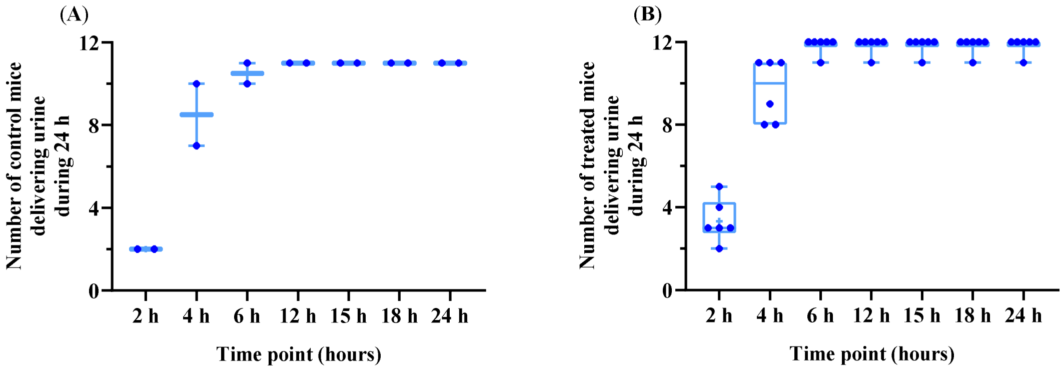

Figure S2. Percentile plots of the median ± IQR number of mice delivering urine during the intestinal permeability (IP) test after the oral administration of iohexol. Urine samples were collecting seven times over a 24–h period (at 2, 4, 6, 12, 15, 18, and 24 h). For each IP test a maximum of (**A**) 11 control or (**B**) 12 treated mice were used. On W5 the group of male mice was 11. The horizontal line in the box is the median (50% percentile), and the upper and lower limits of the box indicate the 75% upper and 25% lower quartiles, respectively. The limits of the upper and lower vertical lines represent the maximum and minimum data values, respectively. Dots represent each IP tests.

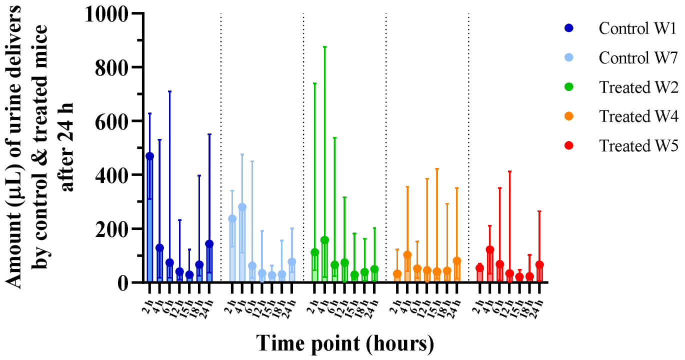

Figure S3. Profile of the median ± IQR volume of mouse urine (µL) collected at seven time points during a 24-h period from control and treated mice after the oral administration of iohexol.

Table S1. Number of mice delivering urine, and the median ± IQR volume of mouse urine, during a total of 8 IP tests at seven time points over a 24-h period in control (n = 11; W1 and W7) and treated mice (n = 23-24; W2, W4, and W5) after the oral administration iohexol.

Time point (hours)	Number of mice (and percentage of all mice) that delivered urine during altogether 8 IP tests	Median ± IQR Volume (µL)
2	23 (24.73%)	80.00 ± 87.00
4	73 (78.50%)	127.00 ± 129.00
6	76 (81.72%)	59.50 ± 70.00
12	82 (88.17%)	40.00 ± 113.25
15	56 (60.22%)	33.00 ± 38.50

18	67 (70.04%)	37.00 ± 46.00
24	76 (81.72%)	71.50 ± 92.75

Table S2. Urinary excretion of iohexol (%) in control and treated groups during a 24-h period after the oral administration of iohexol

Control, iohexol (%)[1]	Treated, iohexol (%)[1]	p-value, one-way ANOVA[2]
0.38 ± 0.36	0.44 ± 0.42	0.223

Control W1, iohexol (%)[1]	Control W7, iohexol (%)[1]	Treated W2, iohexol (%)[1]	Treated W4, iohexol (%)[1]	Treated W5, iohexol (%)[1]	p-value, one-way ANOVA[2]
0.38 ± 0.37	0.37 ± 0.34	0.24 ± 0.41	0.54 ± 0.35	0.55 ± 0.43	***

[1] Results are expressed as means ± SD percentage of iohexol on a base-10 log scale (Log10 (1+x)).
[2] Statistically significant difference between groups, ***$p < 0.001$

Table S3. Urinary excretion of iohexol (%) in female and male control and treated mice measured seven times over a 24-h period after the oral administration of iohexol.

Female mice	Control W1, iohexol (%)[1]	Control W7, iohexol (%)[1]	Treated W2, iohexol (%)[1]	Treated W4, iohexol (%)[1]	Treated W5, iohexol (%)[1]	p-value, one-way ANOVA[2]
	0.42 ± 0.39	0.29 ± 0.29	0.30 ± 0.42	0.51 ± 0.35	0.60 ± 0.45	**0.001
Male mice	Control W1, iohexol (%)[1]	Control W7, iohexol (%)[1]	Treated W2, iohexol (%)[1]	Treated W4, iohexol (%)[1]	Treated W5, iohexol (%)[1]	p-value, one-way ANOVA[2]
	0.35 ± 0.36	0.46 ± 0.37	0.19 ± 0.40	0.56 ± 0.35	0.50 ± 0.41	***

[1] Results are expressed as means ± SD percentage of iohexol on a base-10 log scale (Log10 (1+x)).
[2] Statistically significant difference between groups, **$p < 0.01$, ***$p < 0.001$

Table S4. Urinary excretion of iohexol (%) in 24 treated mice measured seven times over a 24-h period after the oral administration of iohexol.

Time point (hours)	Treated W2, iohexol (%)[1]	Treated W4, iohexol (%)[1]	Treated W5, iohexol (%)[1]	p-value, one-way ANOVA[2]
2	0.25 ± 0.15	0.59 ± 0.28	0.64 ± 0.25	*0.016
4	0.35 ± 0.39	0.70 ± 0.29	0.87 ± 0.18	***
6	0.21 ± 0.45	0.33 ± 0.42	0.46 ± 0.53	0.252
12	0.30 ± 0.52	0.50 ± 0.35	0.44 ± 0.44	0.345
15	0.22 ± 0.36	0.59 ± 0.30	0.31 ± 0.26	*0.012
18	0.20 ± 0.32	0.51 ± 0.37	0.36 ± 0.43	0.061
24	0.20 ± 0.46	0.62 ± 0.26	0.68 ± 0.38	***

[1] Results are expressed as means ± SD percentage of iohexol on a base-10 log scale (Log10 (1+x)).
[2] Statistically significant difference between groups, *$p < 0.05$, ***$p < 0.001$

Table S5. Correlation between the urinary excretion of iohexol in 24 h and the excretion measured at seven time points and in cumulative urinary samples over a 24 h period after the oral administration of iohexol to control (W1 and W7; total n = 22) and treated mice (W2, W4, and W5; total n = 71).

FEMALE MICE	IOHEXOL (%)											
Iohexol (%) (0-24 h)	Single samples							Two samples	Three samples	Four samples	Five samples	Six samples
	2 h	4 h	6 h	12 h	15 h	18 h	24 h	2+4 h	2+4+6 h	2+4+6+12 h	2+4+6+12 +15 h	2+3+4+6 +12+15+ 18 h
Correlation coefficient	0.68	0.48	0.43	0.55	0.52	0.57	0.60	0.60	0.70	0.82	0.83	0.87
n, number of mice	9	32	39	38	20	29	36	36	45	46	46	46
p-value	*	**	**	***	*	**	***	***	***	***	***	***

Correlations were calculated using Spearman's rank coefficients where appropriate. Significance levels are expressed as: **p < 0.05, **p < 0.01, ***p < 0.001

MALE MICE	IOHEXOL (%)											
Iohexol (%) (0-24 h)	Single samples							Two samples	Three samples	Four samples	Five samples	Six samples
	2 h	4 h	6 h	12 h	15 h	18 h	24 h	2+4 h	2+4+6 h	2+4+6+12 h	2+4+6+12 +15 h	2+3+4+6 +12+15+ 18 h
Correlation coefficient	0.91	0.58	0.28	0.42	0.48	0.43	0.53	0.85	0.65	0.78	0.86	0.89
n, number of mice	13	41	38	39	27	34	37	13	41	46	47	47
p-value	***	***		**	*	*	***	**	***	***	***	***

Correlations were calculated using Spearman's rank coefficients where appropriate. Significance levels are expressed as: **p < 0.05, **p < 0.01, ***p < 0.001.

Table S6. Scoring system used for the assessment of histological duodenitis in well-oriented sections stained for Ki-67 (immunohistochemistry) or H/E, at sites of maximal damage.

Villus/crypt (V/C) ratio (maximal score 3.0)	Villus cellular infiltration (maximal score 3)	Basal infiltration with neutrophils (maximal score 3)	Total score (maximal score 3+3+3 = 9)

Measurement of the length of 5 crypt (stained epithelium) and 5 villus (unstained epithelium) zones in sections stained for Ki-67, calculation of the means. Score assigned as follows: Score 0 (V/C ratio > 3.00), 0.5 (2.50–3.00), 1.0 (2.00–2.49), 1.5 (1.50–1.99), 2.0 (1.00–1.49), 2.5 (0.50–0.99), 3.0 (< 0.5)	Assessment of 5 villus/crypt units. Score assigned as follows: Score 0 (villus lamina propria diameter < 0.5 x crypt diameter), 1 (0.5-1x), 2 (1-2x), 3 (> 2x)	Count of the number of crypt abscesses. Score assigned as follows: Score 0 (normal crypts), 1 (1-7 crypt abscess(s) per duodenal section), 2 (8-15 crypt abscesses), 3 (>15 crypt abscesses)	Each specimen assigned a composite histological duodenitis score by combining three separate parameters

Table S7. Spearman's rank correlation between the histological score for duodenitis, duodenum weight and small intestinal weight in control and treated mice.

CORRELATION (Spearman's rho, p value[1], n = nº samples)	Histological score for duodenitis - duodenum weight	Histological score for duodenitis - small intestine weight
All mice	r = 0.76, ***< 0.001, n = 27	r = 0.52, **0.009, n = 24
Female mice	r = 0.60, *0.020, n = 15	r = 0.72, **0.010, n = 12
Male mice	r = 0.71, **0.004, n = 15	r = 0.28, 0.377, n = 12

[1] Statistically significant difference between groups, *p < 0.05, **p < 0.01, ***p < 0.001

Table S8. Spearman's rank correlation between the histological score for duodenitis and the ratio duodenum weight/body weight, and small intestinal weight/body weight in control and treated mice.

CORRELATION (Spearman's rho, p value[1], n = nº samples)	Histological score for duodenitis - duodenum weight to body weight ratio	Histological score for duodenitis – small intestinal weight to body weight ratio
All mice	r = 0.73, ***< 0.001, n = 27	r = 0.37, 0.077, n = 24
Female mice	r = 0.70, **0.005, n = 15	r = 0.53, 0.079, n = 12
Male mice	r = 0.77, **0.001, n = 15	r = 0.75, **0.006, n = 12

[1] Statistically significant difference between groups, **p < 0.01, ***p < 0.001

Table S9. Spearman's rank correlation between the urinary excretion of iohexol and the histological score for duodenitis, duodenum weight and small intestinal weight in control and treated mice. Data is showed as cumulative samples (0-2, 0-4, 0-6, 0-12, 0-15, 0-18 and 0-24 h). *p < 0.05, **p < 0.01, ***p < 0.001

	CUMULATIVE SAMPLES (0-2, 0-4, 0-6, 0-12, 0-15, 0-18, 0-24 h), IOHEXOL (%)		
	CORRELATION (Spearman's rho, p value[1], n = nº samples)		
	Iohexol (%) - histological score for duodenitis	Iohexol (%) - duodenum weight	Iohexol (%) - small intestine weight
W4 female mice	0-2h: r = 0.88, *0.036, n = 5<br>0-4 h: r = 0.05, 0.871, n = 12<br>0-6 h: r = - 0.62, 0.016, n = 15<br>0-12 h: r = - 0.36, 0.187, n = 15<br>0-15 h: r = - 0.356, 0.192, n = 15<br>0-18 h: r = - 0.364, 0.182, n = 15<br>0-24 h: r = - 0.004, 0.992, n = 15	0-2h: r = - 0.23, 0.85, n = 5<br>0-4 h: r = - 0.77, **0.005, n = 12<br>0-6 h: r = - 0.88, ***< 0.001, n = 15<br>0-12 h: r = - 0.60, *0.021, n = 15<br>0-15 h: r = - 0.70, **0.005, n = 15<br>0-18 h: r = - 0.64, *0.012, n = 15<br>0-24 h: r = - 0.44, 0.104, n = 15	0-2h: Too few pairs, n = 2<br>0-4 h: r = - 0.83, **0.008, n = 9<br>0-6 h: r = - 0.57, 0.058, n = 12<br>0-12 h: r = - 0.42, 0.170, n = 12<br>0-15 h: r = - 0.40, 0.200, n = 12<br>0-18 h: r = - 0.25, 0.439, n = 12<br>0-24 h: r = - 0.18, 0.583, n = 12

W5<br>female mice	0-2 h: r = 0.81, 0.092, n = 5<br>0-4 h: r = 0.37, 0.264, n = 11<br>0-6 h: r = 0.37, 0.171, n = 15<br>0-12 h: r = 0.33, 0.235, n = 15<br>0-15 h: r = 0.20, 0.474, n = 15<br>0-18 h: r = 0.20, 0.466, n = 15<br>0-24 h: r = 0.30, 0.281, n = 15	0-2h: r = - 0.50, 0.314, n = 7<br>0-4 h: r = - 0.15, 0.653, n = 11<br>0-6 h: r = 0.06, 0.838, n = 15<br>0-12 h: r = 0.07, 0.813, n = 15<br>0-15 h: r = 0.05, 0.874, n = 15<br>0-18 h: r = - 0.12, 0.670, n = 15<br>0-24 h: r = 0.01, 0.972, n = 15	0-2h: r = - 1.00, 0.083, n = 4<br>0-4 h: r = - 0.30, 0.471, n = 8<br>0-6 h: r = - 0.20, 0.523, n = 12<br>0-12 h: r = 0.27, 0.394, n = 12<br>0-15 h: r = 0.26, 0.407, n = 12<br>0-18 h: r = 0.11, 0.737, n = 12<br>0-24 h: r = 0.18, 0.583, n = 12
W4<br>male mice	0-2 h: r = 0.89, **0.008, n = 8<br>0-4 h: r = 0.50, 0.068, n = 14<br>0-6 h: r = 0.42, 0.118, n = 15<br>0-12 h: r = 0.51, 0.052, n = 15<br>0-15 h: r = 0.69, **0.006, n = 15<br>0-18 h: r = 0.67, **0.008, n = 15<br>0-24 h: r = 0.50, 0.060, n = 15	0-2 h: r = 0.77, *0.036, n = 8<br>0-4 h: r = 0.51, 0.068, n = 14<br>0-6 h: r = 0.47, 0.079, n = 15<br>0-12 h: r = 0.57, *0.030, n = 15<br>0-15 h: r = 0.61, *0.018, n = 15<br>0-18 h: r = 0.55, *0.036, n = 15<br>0-24 h: r = 0.52, 0.051, n = 15	0-2h: r = - 0.60, 0.350, n = 5<br>0-4 h: r = 0.0, >0.999, n = 11<br>0-6 h: r = 0.01, 0.991, n = 12<br>0-12 h: r = 0.03, 0.921, n = 12<br>0-15 h: r = 0.06, 0.864, n = 12<br>0-18 h: r = 0.08, 0.817, n = 12<br>0-24 h: r = 0.11, 0.733, n = 12
W5<br>male mice	0-2 h: r = 0.97, ***< 0.001, n = 5<br>0-4 h: r = 0.79, **0.002, n = 13<br>0-6 h: r = 0.67, **0.010, n = 14<br>0-12 h: r = 0.63, *0.019, n = 14<br>0-15 h: r = 0.54, *0.050, n = 14<br>0-18 h: r = 0.43, 0.126, n = 14<br>0-24 h: r = 0.48, 0.087, n = 14	0-2 h: r = 0.92, *0.05, n = 5<br>0-4 h: r = 0.43, 0.143, n = 13<br>0-6 h: r = 0.46, 0.103, n = 14<br>0-12 h: r = 0.41, 0.142, n = 14<br>0-15 h: r = 0.37, 0.193, n = 14<br>0-18 h: r = 0.52, 0.060, n = 14<br>0-24 h: r = 0.32, 0.269, n = 14	0-2h: Too few pairs, n = 2<br>0-4 h: r = - 0.21, 0.560, n = 10<br>0-6 h: r = - 0.21, 0.539, n = 11<br>0-12 h: r = - 0.04, 0.924, n = 11<br>0-15 h: r = - 0.05, 0.903, n = 11<br>0-18 h: r = 0.20, 0.557, n = 11<br>0-24 h: r = - 0.33, 0.327, n = 11
W4 + W5<br>female mice	0-2 h: r = 0.61, 0.119, n = 8<br>0-4 h: r = 0.01, 0.975, n = 20<br>0-6 h: r = - 0.22, 0.261, n = 27<br>0-12 h: r = - 0.12, 0.551, n = 27<br>0-15 h: r = - 0.15, 0.45, n = 27<br>0-18 h: r = - 0.18, 0.376, n = 27<br>0-24 h: r = 0.09, 0.667, n = 27	0-2 h: r = - 0.50, 0.174, n = 9<br>0-4 h: r = - 0.47, 0.037, n = 20<br>0-6 h: r = - 0.40, 0.039, n = 27<br>0-12 h: r = - 0.28, 0.157, n = 27<br>0-15 h: r = - 0.32, 0.101, n = 27<br>0-18 h: r = - 0.37, 0.054, n = 27<br>0-24 h: r = - 0.19, 0.365, n = 27	0-2h: r = - 0.97, 0.006, n = 6<br>0-4 h: r = - 0.53, 0.030, n = 17<br>0-6 h: r = - 0.23, 0.290, n = 24<br>0-12 h: r = - 0.10, 0.627, n = 24<br>0-15 h: r = - 0.10, 0.632, n = 24<br>0-18 h: r = - 0.09, 0.682, n = 24<br>0-24 h: r = 0.03, 0.874, n = 24
W4 + W5<br>male mice	0-2 h: r = 0.83, **0.006, n = 10<br>0-4 h: r = 0.58, **0.003, n = 24<br>0-6 h: r = 0.55, **0.004, n = 26<br>0-12 h: r = 0.54, **0.005, n = 26<br>0-15 h: r = 0.58, **0.002, n = 26<br>0-18 h: r = 0.52, **0.007, n = 26<br>0-24 h: r = 0.39, *0.047, n = 26	0-2 h: r = 0.77, *0.014, n = 10<br>0-4 h: r = 0.34, 0.107, n = 24<br>0-6 h: r = 0.38, 0.058, n = 26<br>0-12 h: r = 0.43, *0.030, n = 26<br>0-15 h: r = 0.41, *0.039, n = 26<br>0-18 h: r = 0.42, *0.034, n = 26<br>0-24 h: r = 0.29, 0.151, n = 26	0-2h: r = - 0.36, 0.422, n = 7<br>0-4 h: r = - 0.15, 0.526, n = 21<br>0-6 h: r = - 0.12, 0.600, n = 23<br>0-12 h: r = - 0.03, 0.889, n = 23<br>0-15 h: r = - 0.05, 0.836, n = 23<br>0-18 h: r = - 0.04, 0.872, n = 23<br>0-24 h: r = - 0.09, 0.669, n = 23
All W4 + W5<br>female and<br>male mice	0-2 h: r = 0.69, **0.006, n = 15<br>0-4 h: r = 0.28, 0.077, n = 41<br>0-6 h: r = 0.16, 0.266, n = 50<br>0-12 h: r = 0.22, 0.130, n = 50<br>0-15 h: r = 0.25, 0.085, n = 50<br>0-18 h: r = 0.21, 0.148, n = 50<br>0-24 h: r = 0.29, *0.045, n = 50	0-2 h: r = 0.32, 0.231, n = 16<br>0-4 h: r = - 0.025, 0.877, n = 41<br>0-6 h: r = - 0.050, 0.721, n = 50<br>0-12 h: r = 0.06, 0.660, n = 50<br>0-15 h: r = 0.051, 0.72, n = 50<br>0-18 h: r = 0.04, 0.78, n = 50<br>0-24 h: r = 0.10, 0.484, n = 50	0-2h: r = - 0.47, 0.105, n = 13<br>0-4 h: r = - 0.26, 0.110, n = 38<br>0-6 h: r = - 0.15, 0.305, n = 47<br>0-12 h: r = - 0.02, 0.890, n = 47<br>0-15 h: r = - 0.01, 0.952, n = 47<br>0-18 h: r = 0.04, 0.806, n = 47<br>0-24 h: r = 0.08, 0.572, n = 47

[1] Statistically significant difference between groups, *p < 0.05, **p < 0.01, ***p < 0.001
